# Pre-stroke Physical Inactivity and Stroke Severity in Male and Female Patients

**DOI:** 10.3389/fneur.2022.831773

**Published:** 2022-03-11

**Authors:** Pegah Salmantabar, Tamar Abzhandadze, Adam Viktorisson, Malin Reinholdsson, Katharina S. Sunnerhagen

**Affiliations:** ^1^Institute of Neuroscience and Physiology, The Sahlgrenska Academy, University of Gothenburg, Gothenburg, Sweden; ^2^Department of Occupational Therapy and Physiotherapy, Sahlgrenska University Hospital, Gothenburg, Sweden; ^3^Rehabilitation Medicine, Neurocare, Sahlgrenska University Hospital, Gothenburg, Sweden

**Keywords:** sex, sedentary behavior, physical activity, ischemic stroke, age groups, sex differences

## Abstract

**Introduction:**

Females experience more severe ischemic strokes than do males. A higher pre-stroke physical activity level is associated with less severe stroke. The primary aim of this study was to explore the association between pre-stroke physical inactivity and stroke severity in male and female patients.

**Methods:**

This was a retrospective, registry-based study. The data were retrieved from two stroke registries from 2014 to 2019. The primary explanatory variable was physical activity level before the stroke, assessed using the Saltin-Grimby Physical Activity Level Scale. The outcome was moderate to severe stroke at hospital admission, assessed using the National Institutes of Health Stroke Scale (NIHSS). A moderate to severe stroke was defined as a NIHSS score of ≥6. Binary logistic regression analysis was performed to explore if physical inactivity before the stroke could explain stroke severity in male and female patients.

**Results:**

In total, we included 4,535 patients with ischemic stroke. Female patients (*n* = 2,145) had a mean age of 76 years, 35% had a moderate to severe stroke, and 64% were physically inactive pre-stroke. Male patients (*n* = 2,390) had a mean age of 72 years, 25% had a moderate to severe stroke, and 49% were physically inactive pre-stroke. Physical inactivity was associated with higher odds for moderate to severe stroke in both sexes (females' odds ratio [OR], 2.7, 95% confidence interval [CI]: 2.2–3.3, *p* < 0.001 and males' OR, 2.06, 95% CI: 1.7–2.5, *p* < 0.001). The association remained significant in the adjusted models.

**Conclusions:**

Physically inactive females and males had higher odds of experiencing a moderate to severe stroke. However, the OR of female patients was somewhat higher than that of male patients.

## Introduction

There are sex differences in stroke severity. Several studies have indicated that females experience greater stroke severity than do males (1–5). A recurring possible explanation for sex differences in stroke severity is that females have a longer lifespan than males and are approximately 5 years older at stroke onset (1, 4, 6–8). For individuals aged younger than 85 years, males have a higher risk of ischemic stroke than females; however, for those aged older than 85 years, females have a higher risk than males, and a 15% higher stroke mortality compared with age-matched males (9). Moreover, there is a sex difference in the risk factor profile (10), where females who have a first stroke predominantly have arterial hypertension and cardioembolic diseases, whereas males more commonly present with alcohol overuse, are smokers, and have a history of arterial peripheral disease (6). Pre-stroke physical activity (PA) level is associated with lower severity of stroke and better outcomes (11, 12). In a review, six studies reported that patients with higher pre-stroke PA had less severe strokes, whereas two studies found no association (12). A meta-analysis reported that a higher pre-stroke PA level may be more important for females than males to reduce stroke risk (13). In another study, the effect of pre-stroke PA on minor stroke was examined. Results indicated that regardless of age group only light and moderate PA were protective against more severe stroke (14). Although several studies have shown associations between pre-stroke PA and stroke severity, investigations regarding sex differences are limited.

PA promotes health for people of all ages (15). Physical inactivity is a global problem, with one-quarter of the worldwide population being insufficiently physically active (16). Well-established benefits of regular PA are reduced risk of ischemic heart disease, diabetes, and stroke (16, 17). PA is defined as any bodily movement produced by the skeletal muscles that generates energy and can be occupational, sports, conditioning, household, or other activities (18). Depending on sex and age, there appear to be differences in PA; intensity and frequency of PA decline with age in both sexes; however, females exhibit a faster decline (19). Among older people (aged > 65 years), males report higher levels of PA than do females (19, 20). In another study in an older age group (aged 60–75 years), it was revealed that females had a lower frequency, shorter duration, and lighter intensity of leisure-time PA than those of males (21). In a self-reported PA questionnaire study conducted by the National Public Health Survey in Sweden in 2018, females in the younger age group (i.e., 30–44 years) were less physically active than males (64 vs. 70%). In contrast, the opposite was observed in the middle-age group (i.e., 45–64 years; 65 vs. 60%). Furthermore, in the oldest age group (65–84 years) no sex difference was reported (54% for both sexes) (22).

It is known that PA affects several risk factors for stroke and that PA habits as well as the risk factor profile differs between males and females. Additionally, associations between pre-stroke PA and a milder stroke severity have been reported in several studies, but with no study reporting associations for males and females separately, although several studies have adjusted for sex and age. Thus, the primary aim of this study was to explore the association between pre-stroke physical inactivity and stroke severity in male and female patients with ischemic stroke. The secondary aim was to explore sex differences in different age groups in relation to pre-stroke physical inactivity and stroke severity.

## Materials and Methods

### Study Design and Sample

This cross-sectional and retrospective study was conducted as part of the Physical Activity Pre-Stroke in Gothenburg (PAPSIGOT) project (11). Patients who had been admitted to a stroke unit at three sites of Sahlgrenska University hospital were enrolled in the study. The hospitals provide emergency and basic care for the Gothenburg region, which has ~850,000 inhabitants, and offer specialized care for Western Sweden, which has ~1.7 million inhabitants (23). Patients were included if they had experienced an ischemic stroke (I63 according to the International Classification of Diseases (ICD-10), were admitted to a stroke unit from November 1, 2014, to June 30, 2019, had data available on pre-stroke PA and stroke severity at admission, and were aged ≥ 18 years at stroke onset.

### Ethics Statement

The study was approved by the Regional Ethical Board of Gothenburg (approved 4 May 2016; registration number 346-16 and amendment approved 14 May 2020; registration number 2020-01668). Written informed consent for participation was not required. According to the Swedish Data Protection Authority, the handling of data generated within the framework of quality registries is exempt from the general rule requiring written informed consent from patients. Furthermore, the Personal Data Act (Swedish law #1998:204, issued April 29, 1998) allows data from medical charts to be collected for clinical purposes and quality control without written informed consent. Thus, the Declaration of Helsinki was not relevant to this project, which was based on data that were generated within quality registries. The collection and handling of data in this study followed the General Data Protection Regulation in Sweden (2018).

### Procedure

The data were retrieved from three registries: Väststroke, Riksstroke, and Statistics Sweden (SCB). The registries were merged by statisticians at Riksstroke and SCB using personal identification numbers. Thereafter, personal identification numbers were replaced with serial numbers. SCB held the code key. The received data file was pseudonymized.

Väststroke is a quality register for stroke in Gothenburg, Sweden, and all stroke units register data on the register. Väststroke contains information on pre-stroke PA and stroke severity at admission, which are assessed using the Saltin-Grimby Physical Activity Level Scale (SGPALS) and the National Institutes of Health Stroke Scale (NIHSS) (23), respectively. Pre-stroke PA was assessed by physiotherapists working at the stroke units. Patients were asked about their PA on their first encounter with the physiotherapist. Stroke severity at admission was assessed by physicians. In cases with missing observations for SGPALS and NIHSS in the Väststroke register, assessments were retrieved from medical records when possible. Riksstroke is the national quality register for stroke care in Sweden (24). The coverage rate of acute stroke cases was 89% in 2019 (25). Riksstroke comprises information on patients' pre-hospital status, comorbidities, and medical treatment. Data in the Riksstroke register were recorded by trained nurses working at the stroke units. SCB covers the population statistics in Sweden. For this study, data on patients' education and country of birth were retrieved.

### Variables

Stroke severity at admission was assessed using the NIHSS (26). The NIHSS score ranges from 0–42 points, with a higher score indicating more severe stroke. In this study, stroke severity was defined as mild (0–5), moderate (6–14), severe (15–24), and very severe (≥25) (27). The NIHSS was dichotomized for binary logistic regression analyses. To balance the distribution of the data between classes, stroke severity was defined as mild stroke (0–5) and moderate to severe stroke (6–42) (11).

Pre-stroke PA over the past year was assessed using the SGPALS (28). The SGPALS has four levels: (1) physical inactivity, (2) some PA for at least 4 h/week (light PA), (3) regular PA and training for at least 2–3 h/week (moderate PA), and (4) regular hard physical training for competitive sports several times per week (vigorous PA) (29). As the primary explanatory variable SGPALS was dichotomized into physically inactive (level 1) and physically active patients (levels 2–4) to ensure balanced groups in the binary logistic regression models (11).

Other variables analyzed included patients' sociodemographic characteristics, comorbidities, stroke-related treatments, and outcomes. To enable group comparisons by stratifications, age was stratified into four groups: 18–64, 65–74, 75–84, and ≥ 85 years (4); education levels were defined as pre-upper secondary school (≤ 9 years), upper secondary school (10–12 years), and higher education, such as post-secondary education and postgraduate education (≥13 years); country of birth was defined as Sweden and outside of Sweden (because several participants were born outside of Sweden).

The risk factor index for ischemic stroke was created by grouping variables associated with an increased risk of having an ischemic stroke. In a previous study, the comorbidity burden was analyzed in pre-stroke patients with groupings of different variables (30). In the current study, the variable included conditions such as previous stroke, diabetes, smoking, and atrial fibrillation (AF) (31). The aggregated score of the risk factor index ranged from 0 to 4, where 0 indicates that the patient has no risk factors. Because previous transient ischemic attack (TIA) has been associated with lower stroke severity, it was not included in the risk factor index variable (32).

### Statistical Analysis

Dropout analyses (included and excluded patients) and comparisons between male and female patients were performed using the chi-squared test (χ^2^) for nominal variables and the Mann–Whitney *U-*test for continuous variables. Correlations between variables were studied by stratifying the data according to patients' sex. Spearman's rank-order correlation (r_s_) was used for ordinal and scale variables, and the Phi coefficient was used for nominal variables. Correlation coefficients were interpreted as small (< ± 0.39), medium (± 0.40 to ± 0.69), and large (≥ ± 0.70) (33).

Binary logistic regression analyses were conducted to determine if pre-stroke physical inactivity could explain stroke severity in male and female patients. The outcome was moderate to severe stroke, which was defined as an NIHSS score of ≥6. The primary explanatory variable was pre-stroke physical inactivity defined as SGPALS level 1. Other explanatory variables were selected according to the clinical experience of the authors as well as previous literature (8, 34) and comprised age (four strata), previous TIA (yes/no), risk factor index (continuous), education (three strata), country of birth (Sweden, yes/no), and living alone prior to the stroke (yes/no).

Assumptions of the binary logistic regressions were explored prior to model building. Correlation coefficients between variables were explored, and variables with a correlation coefficient ≥ ± 0.7 were interpreted as having multicollinearity and were thus not included in the same regression model (33). Crosstables were explored between the outcome variable and all categorical explanatory variables for testing the assumption of 10 observations per outcome category.

Three binary logistic regression models were built, stratified by sex. The first univariable model explored the raw association between pre-stroke PA and stroke severity. The second model included age group with pre-stroke PA because it is known that older age is related to physical inactivity and greater stroke severity. The third model included all explanatory variables.

The results were evaluated as follows: at the variable level, we reported β coefficients with standard errors (SE), odds ratios (ORs) and 95% confidence intervals (CI), and *p*-values. The models were evaluated using the Hosmer and Lemeshow test (*p* > 0.05 indicates a good fit), the Omnibus test (*p* ≤ 0.05 was desirable), and area under the receiver operating characteristic curve (AUC; a value of ≤ 0.5 indicated poor performance). The explained variance of the models was determined using Cox and Snell's *R*^2^ and Nagelkerke's *R*^2^ tests (higher values were desirable).

All statistical tests were two-tailed with an alpha level of 5%. The SPSS Statistics (IBM Corp. IBM SPSS Statistics for Windows, Version 27.0. Armonk, NY) was used for all statistical analyses (35).

## Results

**Figure 1:**
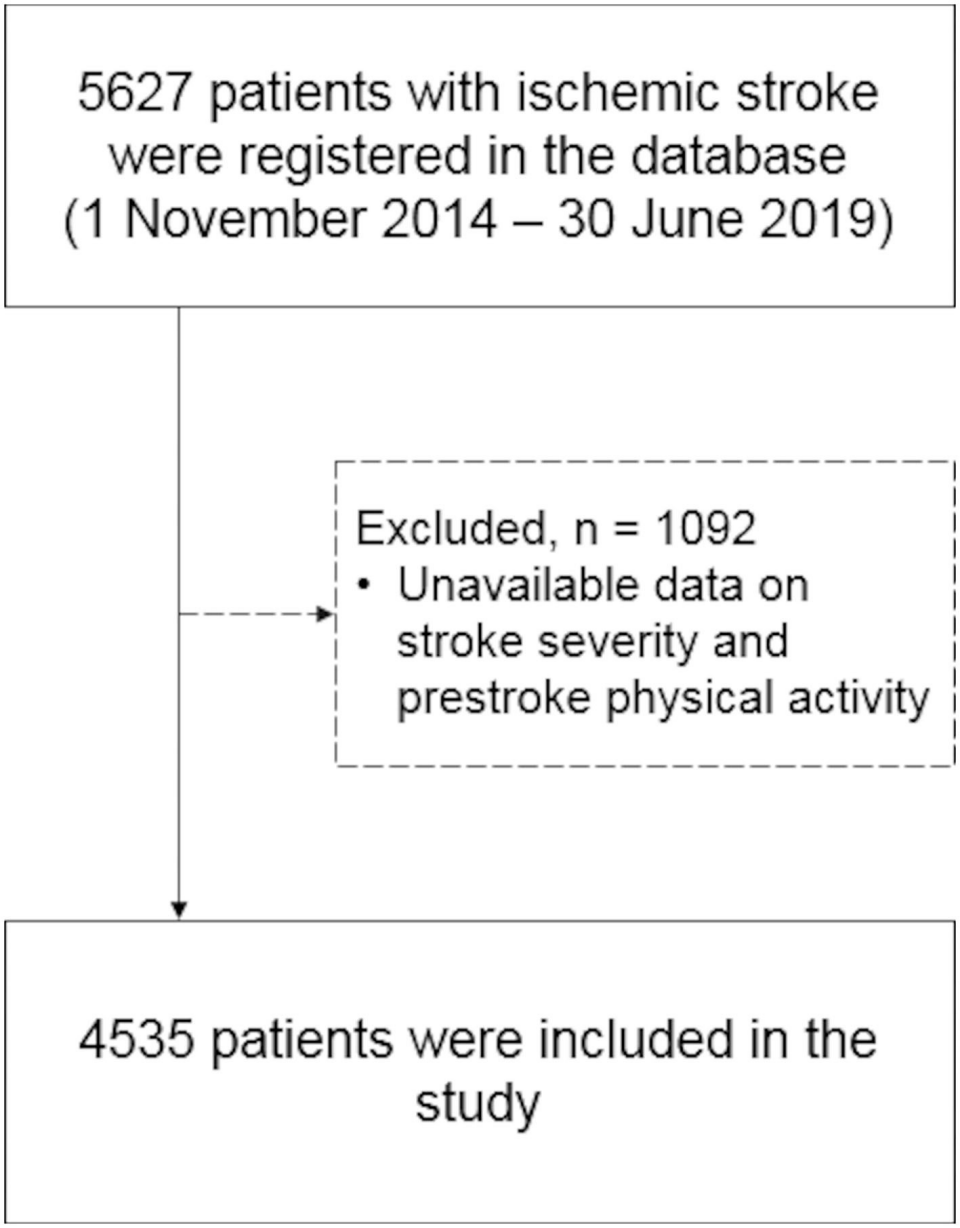
Flow chart of the study participants with ischemic stroke.

A total of 4,535 patients were included in the study from the data file that comprised 5,627 patients. The dropout analyses did not show significant differences between the included (*n* = 4,535) and excluded patients (*n* = 1,092) for sex (*p* = 0.765) or age (*p* = 0.164) (Figure 1).

### Patient Characteristics

Detailed information on the study sample is presented in Table 1. Briefly, there were 2,390 (52.7%) males and 2,145 (47.3%) females with mean (± standard deviation [SD]) ages of 72 years (13.1 years) and 76 years (14.0 years), respectively. Almost half (48.8%) of the males, and 63.9% of the females were physically inactive before the stroke. Median NIHSS scores were 2 and 3 for male and female patients, respectively. A higher proportion of female patients than male patients were physically inactive prior to stroke (*p* < 0.001) and had moderate to severe stroke (*p* < 0.001; Table 1).

**Table 1:** Descriptive characteristics of study participants (*n* = 4,535, ischemic stroke) stratified by sex.

	**Overall (*****n*** **=** **4,535)**		**Male (*****n*** **=** **2,390)**		**Female (*****n*** **=** **2,145)**		***P*-value**
**Characteristics**	***n***	**(%)**	***n***	**(%)**	***n***	**(%)**	
**Age, years**							<0.001
18–64	991	(21.9)	633	(26.5)	358	(16.7)	
65–74	1,101	(24.3)	694	(29.0)	407	(19.0)	
75–84	1,342	(29.6)	670	(28.0)	672	(31.3)	
≥85	1,101	(24.3)	393	(16.4)	708	(33.0)	
**Living alone prior to stroke**							<0.001^#^
Yes	2,206	(49.8)	903	(38.6)	1,303	(62.3)	
Missing	106	(2.3)	51	(2.1)	55	(2.6)	
**Previous transient ischemic attack**							0.412^#^
Yes	334	(7.5)	169	(7.2)	165	(7.9)	
Missing	107	(2.4)	54	(2.3)	3	(2.5)	
**Previous stroke**							0.043^#^
Yes	506	(11.3)	288	(12.3)	218	(10.3)	
Missing	76	(1.7)	41	(1.7)	35	(1.6)	
**Atrial fibrillation**							0.016^#^
Yes	799	(17.9)	390	(16.6)	409	(19.4)	
Missing	74	(1.6)	40	(1.7)	34	(1.6)	
**Diabetes**							<0.001^#^
Yes	878	(19.7)	524	(22.3)	354	(16.8)	
Missing	73	(1.6)	38	(1.6)	35	(1.6)	
**Smoking**							0.066^#^
Yes	570	(14.6)	326	(15.5)	244	(13.5)	
Missing	624	(13.8)	292	(12.2)	332	(15.5)	
**Country of birth**							0.924^#^
Sweden	3,618	(80.3)	1,907	(80.3)	1,711	(80.2)	
Abroad	889	(19.7)	467	(19.7)	422	(19.8)	
Missing	28	(0.6)	16	(0.7)	12	(0.6)	
**Education level**							<0.001
Pre-upper secondary school, ≤ 9 years	1,529	(34.7)	689	(29.6)	840	(40.4)	
Upper secondary school, 10–12 years	1,755	(39.8)	966	(41.4)	789	(38.0)	
Higher education, ≥13 years	1,125	(25.5)	676	(29.0)	449	(21.6)	
Missing	126	(2.8)	59	(2.5)	67	(3.1)	
**Stroke severity, NIHSS score**							<0.001
Mild stroke (0–5)	3,180	(70.1)	1,781	(74.5)	1,399	(65.2)	
Moderate stroke (6–14)	889	(19.6)	407	(17.0)	482	(22.5)	
Severe and very stroke (15–36)	466	(10.3)	202	(8.5)	264	(12.3)	
**Reperfusion treatments (thrombolysis and/or thrombectomy)**							0.111
Yes	875	(19.7)	482	(20.6)	393	(18.7)	
Missing	83	(1.8)	45	(1.9)	38	(1.8)	
**Level of pre-stroke PA (SGPALS)**							<0.001
Physically inactive	2,536	(55.9)	1,166	(48.8)	1,370	(63.9)	
Light PA	1,734	(38.2)	1,035	(43.3)	699	(32.6)	
Moderate PA	255	(5.6)	180	(7.5)	75	(3.5)	
Vigorous PA	10	(0.2)	9	(0.4)	1	(0.0)	
**Blood pressure-lowering medication**							<0.001^#^
Yes	2,753	(61.8)	1,389	(59.2)	1,364	(64.6)	
Missing	77	(1.7)	43	(1.8)	34	(1.6)	
**Lipid-lowering medication**							0.028^#^
Yes	1,165	(26.2)	646	(27.5)	519	(24.6)	
Missing	80	(1.8)	43	(1.8)	37	(1.7)	

*Group comparisons were performed using ^#^χ^2^ and Mann-Whitney U-tests. NIHSS, national institutes of health stroke scale; PA, physical activity; SGPALS, saltin-grimby physical activity level scale*.

### Association Between Pre-stroke Physical Inactivity and Stroke Severity

**Figure 2:**
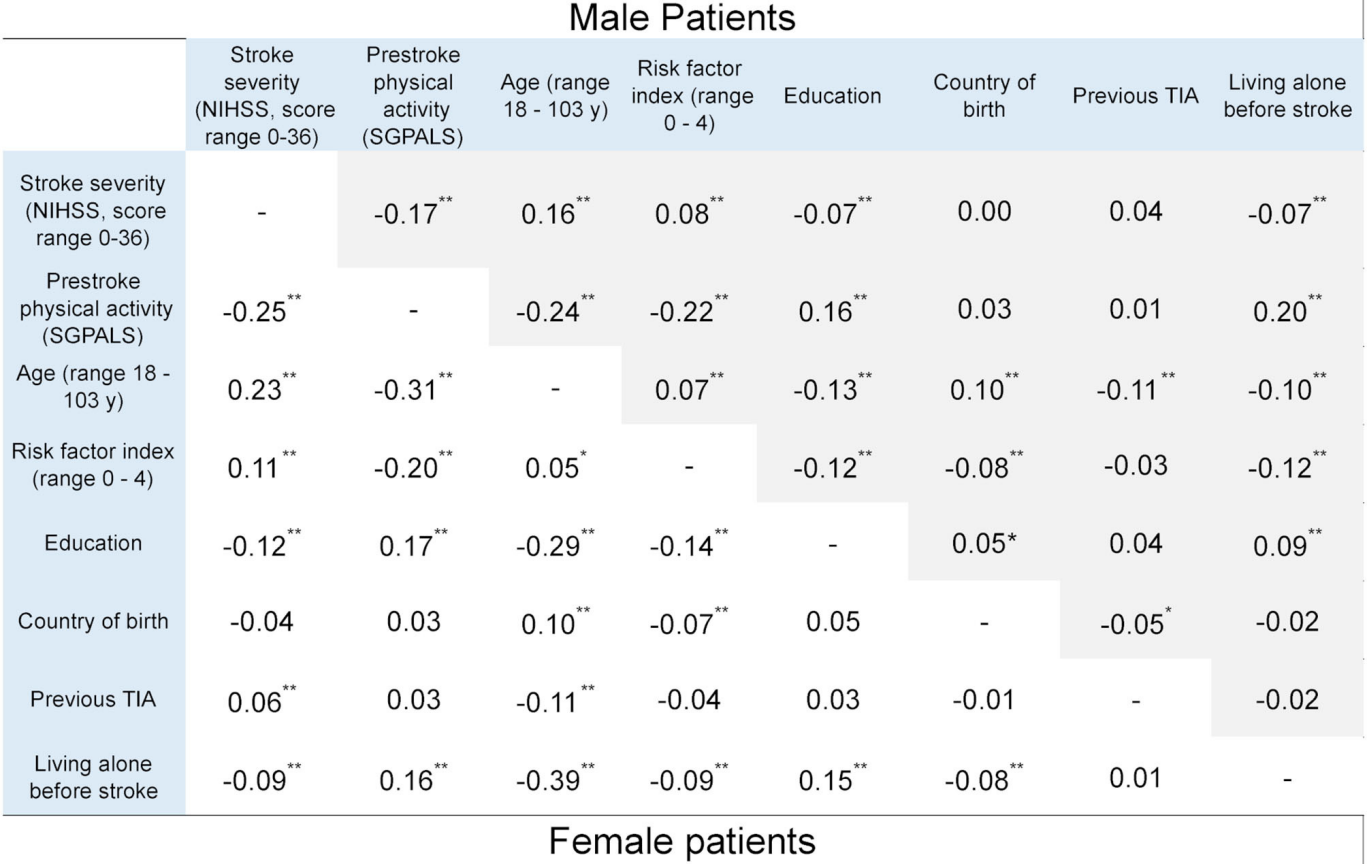
Correlation coefficients between explanatory variables and the outcome variable, stratified by sex (*p < 0.05 and **p < 0.01). Statistics: phi correlation coefficient for binary variables and Spearman correlation coefficient for ordinal variables. NIHSS, national institutes of health stroke scale; SGPALS, saltin-grimby physical activity level scale (range 1–4; 1 is physically inactive); TIA, transient ischemic attack.

Pre-stroke PA, measured using the SGPALS, was negatively correlated with stroke severity, as measured using the NIHSS, in both male and female patients. A lower level of PA was correlated with greater stroke severity. However, the strength of correlation was small: *r*_*s*_ −0.17 (*p* < 0.01) and *r*_*s*_ −0.25 (*p* < 0.01) in male and female patients, respectively (Figure 2). A lower level of PA was correlated with older age in both sexes (males: rs −0.24 [*p* < 0.01] and females rs −0.31 [*p* < 0.01]).

Univariable binary logistic regression models showed that physical inactivity was significantly associated with moderate to severe stroke in both male and female patients, with an OR of 2.06 (95% CI: 1.71–2.50) and 2.72 (95% CI: 2.22–3.33), respectively (Table 2).

**Table 2:** Results of the univariable binary logistic regression analyses stratified by sex, showing the explanatory value of pre-stroke physical inactivity in relation to moderate to severe stroke.

	**Male patients**				**Female patients**			
	**β (SE)**	**OR (95% CI)**	***P*-value**	**AUC**	**β (SE)**	**OR (95% CI)**	***P*-value**	**AUC**
Pre-stroke physical inactivity (SGPALS, 1)	0.72 (0.1)	2.06 (1.71–2.50)	<0.001	0.61	1 (0.1)	2.72 (2.22–3.33)	<0.001	0.65

*SE, standard error; OR, odds ratio; CI, confidence intervals; AUC, area under the receiver operating characteristic curve; SGPALS, saltin-grimby physical activity level scale. Model evaluation for male/female patients: Hosmer and Lemeshow test, 0/0; Omnibus test, <0.001/<0.001; Cox and Snell's R^2^, 0.02/0.05; Nagelkerke's R^2^, 0.04/0.06*.

Pre-stroke physical inactivity remained a significant explanatory variable when it was adjusted for patients' age. However, the OR was lower than that of the univariable model (Table 3). Physically inactive male patients had 1.9 times higher odds of experiencing a moderate-severe stroke (OR: 1.90, 95% CI: 1.57–2.30) and physically inactive female patients had 2.3 times higher odds of experiencing a moderate-severe stroke (OR: 2.30, 95% CI: 1.86–2.84).

**Table 3:** Results of the multivariable binary logistic regression analyses stratified by sex, showing the explanatory value of pre-stroke physical inactivity adjusted for age in relation to moderate to severe stroke.

	**Male patients**			**Female patients**		
	**β (SE)**	**OR (95% CI)**	***P*-value**	**β (SE)**	**OR (95% CI)**	***P*-value**
Pre-stroke physical inactivity (SGPALS, 1)	0.64 (0.1)	1.90 (1.57–2.30)	**<0.001**	0.83 (0.1)	2.30 (1.86–2.84)	**<0.001**
Age ≤ 64 years, Ref.			**<0.001**			**<0.001**
65–74 years	0.13 (0.1)	1.14 (0.87–1.48)	0.34	0.42 (0.2)	1.52 (1.09–2.12)	**0.014**
75–84 years	0.29 (0.1)	1.33 (1.03–1.73)	**0.031**	0.36 (0.2)	1.44 (1.06–1.95)	**0.02**
85+ years	0.59 (0.1)	1.79 (1.34–2.4)	**<** **0.001**	0.93 (0.2)	2.54 (1.88–3.43)	**<0.001**

*Ref, Reference; SE, standard error; OR, odds ratio; CI, confidence intervals; AUC, area under the receiver operating characteristic curve; SGPALS, saltin-grimby physical activity level scale. Bold text indicates statistical significance. Model evaluation for male/female patients. Hosmer and Lemeshow test, 0.66/0.86; Omnibus test, <0.001/ <0.001; Cox and Snell's R^2^, 0.03/0.07; Nagelkerke's R^2^, 0.05/0.09; AUC, 0.61/0.65*.

**Figure 3:**
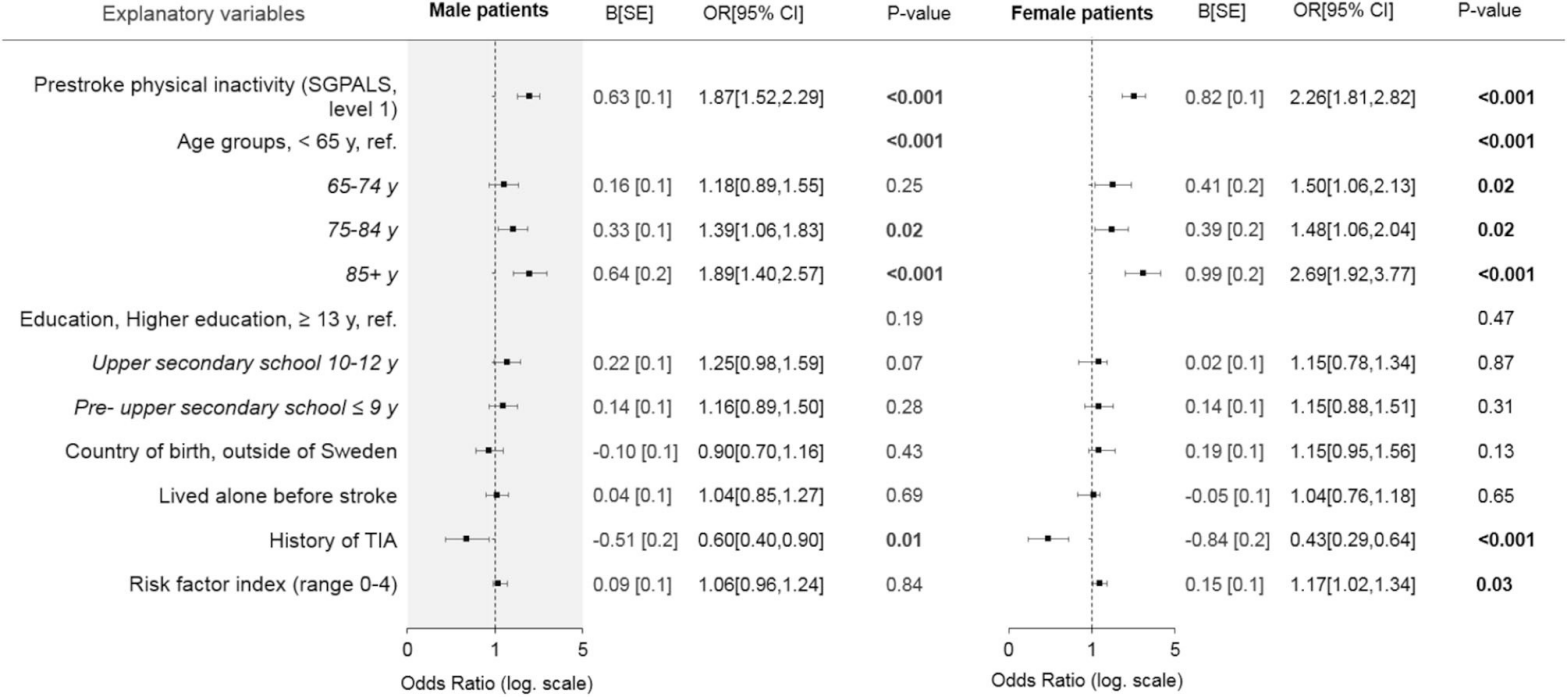
Forest plot showing the results of the multivariable binary logistic regression models stratified by sex, with all explanatory variables in relation to moderate to severe stroke. Bold text indicates statistical significance. SGPALS, saltin-grimby physical activity level scale; TIA, transient ischemic attack. SE, standard error; OR, odds ratio; CI, confidence interval. Model evaluation metrics for male and female patients: Hosmer and Lemeshow test, 0.12/0.83; Omnibus test, <0.001/<0.001; Cox and Snell's *R*^2^, 0.04/0.08; Nagelkerke's *R*^2^, 0.06/0.11; area under the receiver operating characteristic curve, 0.63/0.68.

In the multivariable model that included all explanatory variables, pre-stroke physical inactivity remained a significant explanatory variable for moderate-severe stroke in both sexes (Figure 3). Education level and country of birth were non-significant variables (Figure 3).

## Discussion

This register-based study showed that pre-stroke physical inactivity is associated with severe stroke in both males and females. Physical inactivity was associated with a more severe stroke for both sexes, but with higher values for OR values for females. This result remained after adjusting for other variables. In addition, we found that older patients had higher odds of experiencing moderate to severe stroke, which was observed across both sexes. The highest ORs were observed in the oldest age group (≥85 years) of both sexes, although with higher values in females.

We found that more females than males were physically inactive and older before the onset of stroke, which is representative of the stroke population (19, 20). Significant associations were found between pre-stroke physical inactivity and stroke severity in both the correlation and regression analyses, although effect sizes were low. In the regression model, PA was adjusted for age groups, which slightly increased the effect size of the model. In the multivariable models that included the explanatory variables, the effect sizes were generally low. Stroke severity is a complex outcome and can depend on numerous factors. Although we included several in our analyses, data on other factors were not available in the registries. Reviews have found that cardiac diseases, stroke localization, occlusion level, stroke volume, pre-stroke dependency, pre-stroke institutionalization, and time to hospitalization are related to stroke severity (8, 34).

In the present study, females had more severe strokes than did males, which is in line with previous studies (4, 5). The higher proportion of physical inactivity could be explained by the higher age among the females. In addition, physical inactivity was related to stroke severity with higher OR values in the oldest groups for both sexes. Furthermore, the association was seen in all age groups (65–85+ years) for females, but in two age groups (75–85+ years) for males. Females having at least one risk factor (e.g., smoking, diabetes, previous stroke, or AF) had higher odds of a severe stroke. This is in line with a study where older age, AF, and pre-stroke functional dependency were possible explanations for greater stroke severity in females than males; with a 35% higher risk for females to experience a more severe stroke (8). Another study confirms that males and females differ in the prevalence of stroke risk factors (6). Females with ischemic stroke are more likely to have AF and experience thromboembolic events (5, 36), whereas males are more likely to have a history of diabetes and smoking (5). In the present study, a higher proportion of females were living alone before the stroke, this is consistent with previous findings (4, 5). Living alone is associated with a longer time to hospitalization (5). We found that neither education level nor country of birth were associated with stroke severity. Older patients with lower education have an increased risk of stroke; however, no sex differences were observed (37). Although PA differs depending on geography (15), country of birth was not shown to be an important factor.

### Limitations and Strengths

Self-reported PA, as measured by the SGPALS, can introduce recall bias because patients may experience difficulties in recalling and reporting PA levels. Recall bias was reduced by detailed follow-up questions by assessors and conversations with relatives. Another limitation of the SGPALS is the self-reported data. Although objective measures of PA are always preferred, they may not be feasible in acute stroke situations and large samples. Moreover, there were numerous assessors, which may have increased the risk of assessment bias. However, staff at stroke units have undergone local training and follow hospital routines; moreover, the SGPALS is a commonly used assessment tool that has been used in more than 600,000 subjects in numerous studies, especially in Nordic countries (38). Finally, in our study, stroke severity was measured using the NIHSS, which is a well-validated and widely used neurological stroke scale (26).

In the SGPALS, physical inactivity is defined as <4 h PA per week. In various studies, physical inactivity is referred to as sedentary behavior, and the definition of PA, especially sedentary behavior, is not consistent across previous studies (39). Thus, comparisons between studies are difficult.

Registries comprise consecutively collected data from large samples in clinical settings. Therefore, results from registry-based studies are generalizable across similar clinical settings. In Sweden, health care is tax-financed and available to everyone. In Riksstroke, 86% of the patients admitted to the Sahlgrenska University hospital were registered (25). Although the coverage is high, internal missing data are a common problem in registries, which results in selection bias. However, in this study, there were no significant differences in sex or age between the included and excluded patients. Moreover, the majority of our sample had a mild stroke, which is similar to the general Swedish population, according to the National Stroke Register (4). In addition, females in our sample were 4 years older than males at stroke onset, which is comparable with the national stroke population (4). Thus, our results have good generalizability within a similar context.

Stroke severity at admission is associated with numerous different factors. Further research including standardized measurements, sociodemographic characteristics of patients, and underlying biological mechanisms in various subgroups, such as sex, would contribute to valuable knowledge in this field and public health strategies.

## Conclusion

Physical inactivity before stroke was associated with moderate to severe stroke in both sexes. However, female patients had higher ORs for physical inactivity, and this trend persisted even when physical inactivity was adjusted for age and other covariates. Our results suggest that PA should be encouraged in healthcare and public health sectors.

## Data Availability Statement

The data analyzed in this study is subject to the following licenses/restrictions: according to the Swedish regulations the datasets generated for this study cannot be made publicly available for ethical and legal reasons. Researchers can submit requests for data to the authors (contact: ks.sunnerhagen@neuro.gu.se). Requests to access these datasets should be directed to ks.sunnerhagen@neuro.gu.se.

## Ethics Statement

The study was approved by the Regional Ethical Board of Gothenburg (approved 4 May 2016; registration number 346-16 and amendment approved 14 May 2020; registration number 2020-01668). Written informed consent for participation was not required for this study in accordance with the national legislation and the institutional requirements.

## Author Contributions

PS: conceptualization of the study, analysis and interpretation of the data, and drafting of the manuscript. TA: conceptualization of the study, data analysis, interpretation of the data, and revising the manuscript for intellectual content. AV: acquisition of data and revising the manuscript for intellectual content. MR: acquisition of data, design and conceptualization of the study, and revising the manuscript for intellectual content. KS: design and conceptualization of the study, interpretation of the results, and revising the manuscript for intellectual content. All authors contributed to the article and approved the submitted version.

## Funding

This study was financed by grants from the Swedish Research Council (VR2017-00946), NEURO Sweden, the Swedish Heart and Lung Foundation, the Swedish Brain Foundation, the Swedish state under an agreement between the Swedish government and the county councils, the ALF agreement (ALFGBG-718711 and ALFGBG-877961), the Swedish National Stroke Association, the Local Research and Development Board for Gothenburg and Södra Bohuslän (VGFOUGSB-941553), the Greta and Einar Asker's Foundation, Rune and Greta Almöv's Foundation for Neurological Research, Hjalmar Svensson's Research Foundation, Herbert and Karin Jacobson's Foundation, and Doktor Felix Neubergh's Foundation, Gun and Bertil Stohne's Foundation, and Sahlgrenska University Hospital foundations.

## Conflict of Interest

The authors declare that the research was conducted in the absence of any commercial or financial relationships that could be construed as a potential conflict of interest.

## Publisher's Note

All claims expressed in this article are solely those of the authors and do not necessarily represent those of their affiliated organizations, or those of the publisher, the editors and the reviewers. Any product that may be evaluated in this article, or claim that may be made by its manufacturer, is not guaranteed or endorsed by the publisher.
